# Assessment of Amino Acid/Drug Transporters for Renal Transport of [^18^F]Fluciclovine (*anti*-[^18^F]FACBC) in Vitro

**DOI:** 10.3390/ijms17101730

**Published:** 2016-10-14

**Authors:** Masahiro Ono, Atsumi Baden, Hiroyuki Okudaira, Masato Kobayashi, Keiichi Kawai, Shuntaro Oka, Hirokatsu Yoshimura

**Affiliations:** 1 Research Center, Nihon Medi-Physics Co., Ltd., Chiba 299-0266, Japan; masahiro_ono@nmp.co.jp (M.O.); atsumi_baden@nmp.co.jp (A.B.); hiroyuki_okudaira@nmp.co.jp (H.O.); hirokatsu_yoshimura@nmp.co.jp (H.Y.); 2 Wellness Promotion Science Center, Institute of Medical, Pharmaceutical and Health Science, Kanazawa University, Ishikawa 920-0942, Japan; kobayasi@mhs.mp.kanazawa-u.ac.jp; 3 Division of Health Sciences, Graduate School of Medical Sciences, Kanazawa University, Ishikawa 920-0942, Japan; kei@mhs.mp.kanazawa-u.ac.jp

**Keywords:** fluciclovine, *anti*-FACBC, positron emission tomography, amino acid transporter, drug transporter

## Abstract

[^18^F]Fluciclovine (*trans*-1-amino-3-[^18^F]fluorocyclobutanecarboxylic acid; *anti*-[^18^F]FACBC), a positron emission tomography tracer used for the diagnosis of recurrent prostate cancer, is transported via amino acid transporters (AATs) with high affinity (*K_m_*: 97–230 μM). However, the mechanism underlying urinary excretion is unknown. In this study, we investigated the involvement of AATs and drug transporters in renal [^18^F]fluciclovine reuptake. [^14^C]Fluciclovine (*trans*-1-amino-3-fluoro[1-^14^C]cyclobutanecarboxylic acid) was used because of its long half-life. The involvement of AATs in [^14^C]fluciclovine transport was measured by apical-to-basal transport using an LLC-PK1 monolayer as model for renal proximal tubules. The contribution of drug transporters herein was assessed using vesicles/cells expressing the drug transporters P-glycoprotein (P-gp), breast cancer resistance protein (BCRP), multidrug resistance-associated protein 4 (MRP4), organic anion transporter 1 (OAT1), organic anion transporter 3 (OAT3) , organic cation transporter 2 (OCT2), organic anion transporting polypeptide 1B1 (OATP1B1), and organic anion transporting polypeptide 1B3 (OATP1B3). The apical-to-basal transport of [^14^C]fluciclovine was attenuated by l-threonine, the substrate for system alanine-serine-cysteine (ASC) AATs. [^14^C]Fluciclovine uptake by drug transporter-expressing vesicles/cells was not significantly different from that of control vesicles/cells. Fluciclovine inhibited P-gp, MRP4, OAT1, OCT2, and OATP1B1 (IC_50_ > 2.95 mM). Therefore, system ASC AATs may be partly involved in the renal reuptake of [^18^F]fluciclovine. Further, given that [^18^F]fluciclovine is recognized as an inhibitor with millimolar affinity for the tested drug transporters, slow urinary excretion of [^18^F]fluciclovine may be mediated by system ASC AATs, but not by drug transporters.

## 1. Introduction

[^18^F]Fluciclovine *(trans*-1-amino-3-[^18^F]fluorocyclobutanecarboxylic acid, also known as *anti*-[^18^F]FACBC or Axumin™) is a positron emission tomography (PET) tracer that has been approved by the U.S. Food and Drug Administration for the detection of recurrent prostate cancer since 2016 [1]. Recently, phase II clinical trials of [^18^F]fluciclovine have been simultaneously conducted in Japan for the diagnosis of glioma [2] and primary prostate cancer [3].

[^18^F]Fluciclovine is a synthetic leucine analog that is developed as a next-generation PET tracer to compensate for the high accumulation of [^18^F]fluorodeoxyglucose ([^18^F]FDG) in the brain and urinary tract under physiological conditions [4]. The low urinary excretion of [^18^F]fluciclovine differs from that of many other ^18^F-labeled amino acids such as *O*-(2-[^18^F]fluoroethyl)-l-tyrosine ([^18^F]FET) and a range of tracers that have been preclinically evaluated [5]. Clinical trials have demonstrated that [^18^F]fluciclovine accumulates readily in the liver and pancreas, mildly in the kidneys, and minimally in the brain after administration as an intravenous bolus infusion; it is excreted slowly in the urine for up to 24 hours, but not in bile [6,7]. These results suggest that [^18^F]fluciclovine-PET may have advantages over [^18^F]FDG and [^18^F]FET for imaging tumors in the brain and/or in the pelvis.

Our previous preclinical studies have shown that [^18^F]fluciclovine is not incorporated into proteins [8], and accumulates in cells through amino acid transporters (AATs) that serve as its primary means of transport [8,9,10]. We have also shown that two Na^+^-dependent AAT subtypes (i.e., alanine-serine-cysteine transporter 2 (ASCT2) and sodium-coupled neutral amino acid transporter 2 (SNAT2)) and Na^+^-independent l-type amino acid transporter 1 (LAT1) have high affinities for [^18^F]fluciclovine with *K_m_* values of 92.0, 222.0, and 230.4 μM, respectively [11]. This suggests that AATs may be involved in the whole-body distribution of [^18^F]fluciclovine, because these AATs are expressed in the liver, renal proximal tubule, by the blood-brain-barrier (BBB) [12], and/or tumors [13].

Drug transporters expressed in the intestines, liver, kidneys, and/or at the BBB play an important role in regulating drug absorption, distribution, metabolism, and excretion [14]. Numerous studies have shown that drug transporters influence pharmacokinetics and thus affect drug efficacy and are involved in the occurrence of adverse effects, including those produced by drug-drug interactions. The International Transporter Consortium advocated the assessment of drug-drug interactions in the context of drug transporters in accordance with their significant clinical importance. In particular, three ATP-binding cassette (ABC) transporters—P-glycoprotein (P-gp; MDR1), breast cancer resistance protein (BCRP), and multidrug resistance-associated protein 4 (MRP4)—are important efflux transporters that regulate intestinal absorption, distribution to the brain, and/or excretion in the liver and kidneys for several therapeutic drugs [14]. Three solute carrier (SLC) transporters, organic anion transporter 1 (OAT1), organic anion transporter 3 (OAT3), and organic cation transporter 2 (OCT2), are thought to be involved in transporting anionic (OAT1/3) and cationic (OCT2) drugs from the blood to renal tubules. In addition, two other SLC transporters, organic anion transporting polypeptide 1B1 (OATP1B1) and organic anion transporting polypeptide 1B3 (OATP1B3), are believed to be critical hepatic transporters of anionic drugs [14].

Recent studies have reported that therapeutic drugs that have both amino and carboxyl residues are transported via several transporters. For example, melphalan (an anticancer drug) and gabapentin (antiepilepsy drug) are transported by an AAT (LAT1) [15,16] and drug transporters (P-gp and organic cation/carnitine transporter 1 (OCTN1)) [17,18]. Efflux drug transporters such as P-gp are overexpressed in several tumors, causing resistance to chemotherapeutic drugs [19,20]. Therefore, the contributions of hepatic and renal drug transporters to [^18^F]fluciclovine influx/efflux merit evaluation, because drug transporters such as those described above may be involved in [^18^F]fluciclovine accumulation following [^18^F]fluciclovine administration during PET procedures.

As described above, we have previously reported that AATs are the primary transporters involved in [^18^F]fluciclovine uptake into cells. However, the mechanisms underlying hepatic/renal distribution of [^18^F]fluciclovine by AATs and drug transporters, especially the slow excretion into urine, has not been fully identified. To address this, we assessed the contribution of AATs on renal reabsorption of [^18^F]fluciclovine using the LLC-PK1 monolayer model, which is used to simulate the transport of novel drugs to the proximal tubules [21,22]. Furthermore, the contribution of drug transporters to the hepatic/renal distribution of [^18^F]fluciclovine was investigated.

## 2. Results

### 2.1. Tight Junction Formation in the LLC-PK1 Monolayer Model

**Figure 1:**
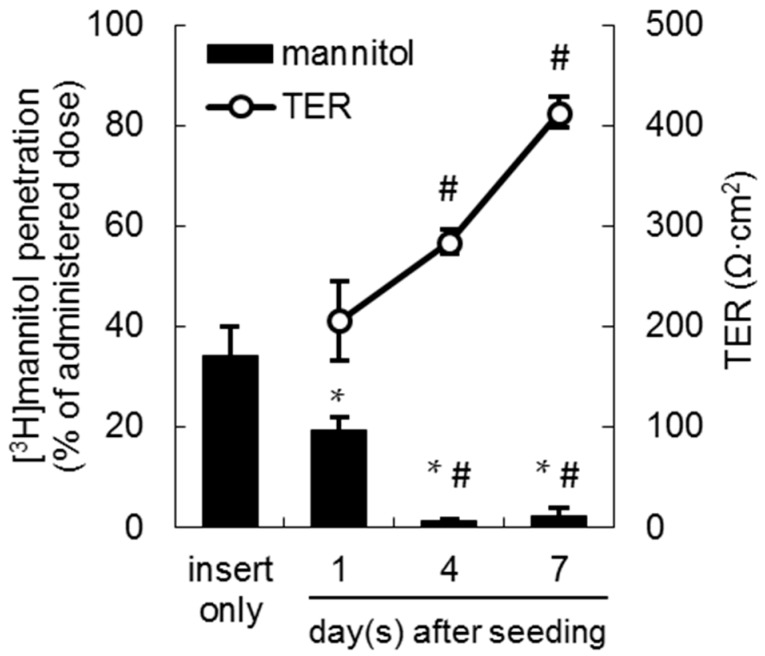
The chronological formation of tight junctions in the LLC-PK1 monolayer model as monitored by the [^3^H]mannitol penetration rate (**left axis**, black bar; *n* = 3–9) and transepithelial electrical resistance (TER, **right axis**, white circle; *n* = 3–4). Each data point represents the mean ± standard deviation (SD). * *p* < 0.05 vs. insert only, # *p* < 0.05 vs. day 1 after seeding.

The LLC-PK1 monolayer model is a well-known model of renal proximal tubules for the estimation of renal transport [21,22]. To validate the formation of tight junction in the LLC-PK1 monolayers, paracellular [^3^H]mannitol penetration, and transepithelial electrical resistance (TER) were measured. As shown in Figure 1, the mean percentage of [^3^H]mannitol permeation was significantly decreased from 19.3% (day 1) to 1.23% (day 4), and reached a plateau at day 7 after seeding (2.29%). In addition, TER values gradually increased to 206, 284, and 413 Ω·cm^2^ at day 1, 4, and 7, respectively (Figure 1). These results suggested that tight junctions were sufficiently formed in the LLC-PK1 monolayers from day 4 after seeding.

### 2.2. Apical-to-Basal Transport of Amino Acid Tracers through the LLC-PK1 Monolayers

**Figure 2:**
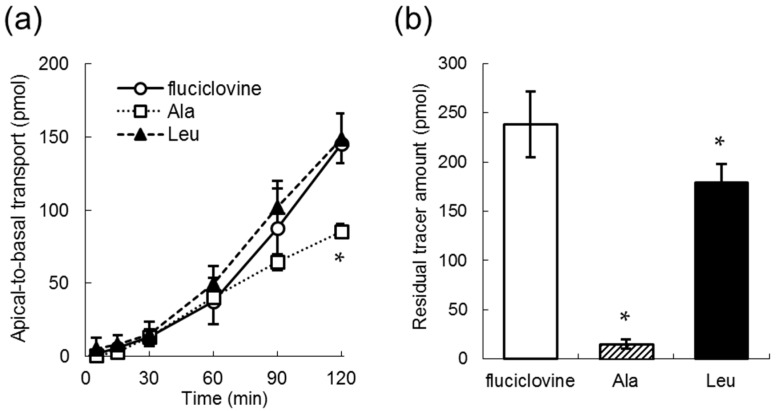
(**a**) Time course of ^14^C-labeled amino acid (10 µM) transport through the LLC-PK1 monolayers after 4–5 days culture (*n* = 3–6); (**b**) the residual amount of intracellular amino acid tracer at 120 min (*n* = 3–6); “fluciclovine”, “Ala”, and “Leu” indicate “[^14^C]fluciclovine”, “l-[^14^C]alanine”, and “l-[^14^C]leucine”, respectively. Each data point represents the mean ± SD. * *p* < 0.05 vs. fluciclovine.

To estimate transcellular amino acid transport through the LLC-PK1 monolayers mimicking renal proximal tubules, apical-to-basal transport was measured using ^14^C-labeled amino acid tracers (10 µM). As shown in Figure 2a, the apical-to-basal transport of each tracer increased in a time-dependent manner. The mean penetration amount of each tracer at 120 min was 145 with [^14^C]fluciclovine (fluciclovine), 85.5 with l-[^14^C]alanine (Ala), and 149 pmol with l-[^14^C]leucine (Leu). In addition, the mean residual radioactivity of [^14^C]fluciclovine in the cells at 120 min was significantly higher than that of Ala and Leu: 238, 15.2, and 179 pmol, respectively (Figure 2b).

### 2.3. The Inhibition of Apical-to-Basal Transport in the LLC-PK1 Monolayers by Amino Acids

**Figure 3:**
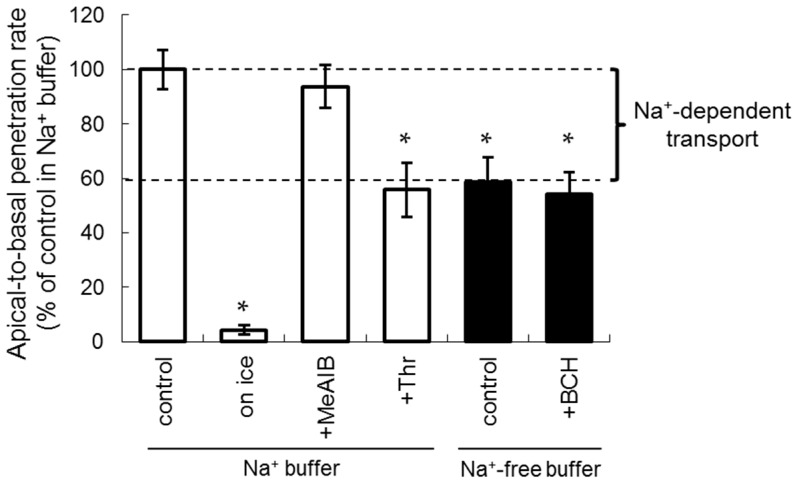
The inhibition profile of [^14^C]fluciclovine (10 μM) apical-to-basal transport using the LLC-PK1 monolayer model under the indicated conditions, i.e., temperature and 1 mM inhibitors in Na^+^ buffer (white bar, *n* = 5–14) or Na^+^-free buffer (black bar, *n* = 9). Each data point represents the mean ± SD. * *p* < 0.05 vs control in Na^+^ buffer.

The inhibition of [^14^C]fluciclovine transport (10 µM) by naturally occurring and synthetic amino acids (1 mM) was assessed using the LLC-PK1 monolayer model. As shown in Figure 3, the apical-to-basal transport rate of [^14^C]fluciclovine in Na^+^-free buffer was 58.8% of the control in Na^+^ buffer, indicating that the contribution of Na^+^-dependent transport was an estimated 41.2% of the total penetration amount (Figure 3; the percentage “control in Na^+^-free buffer” that was subtracted from “control in Na^+^ buffer”). In addition, significant inhibition of apical-to-basal transport was observed in the presence of a combination of Na^+^ and l-threonine (Thr) (the substrate for an Na^+^-dependent system ASC AATs, including ASCT2) to the same extent as that observed in the absence of Na^+^. In contrast, no significant inhibition was observed when using 2-(methylamino)-isobutyric acid (MeAIB, a substrate for the Na^+^-dependent system A AATs, including SNAT2) in the presence of Na^+^, and when using 2-aminobicyclo[2,2,1]heptane-2-carboxylic acid (BCH, a substrate for the Na^+^-independent AAT system L, including LAT1) in the absence of Na^+^. These results suggest that Na^+^-dependent system ASC AATs partly mediate renal [^14^C]fluciclovine transport.

### 2.4. [^14^C]Fluciclovine Transport via ATP-binding Cassette (ABC) Transporters and the Inhibitory Effect of Fluciclovine on ABC Transporters

**Figure 4:**
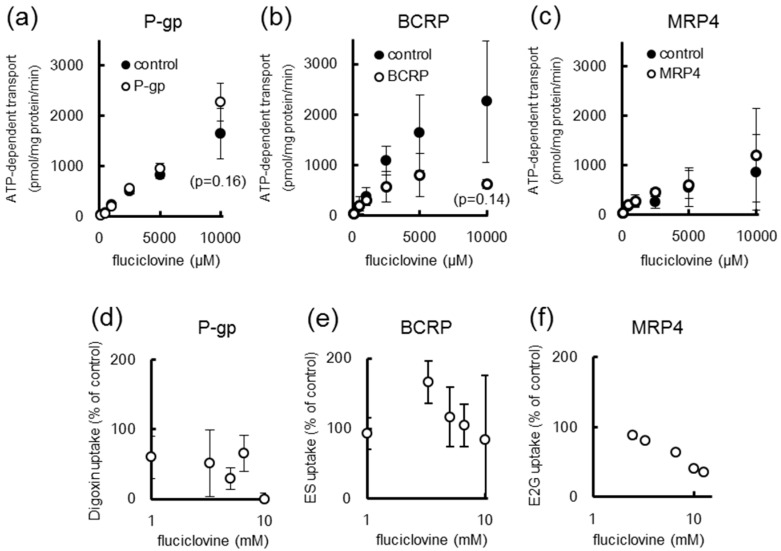
(**a**–**c**): The concentration dependency of [^14^C]fluciclovine in (**a**) P-glycoprotein (P-gp)-; (**b**) breast cancer resistance protein (BCRP)-; and (**c**) multidrug resistance-associated protein 4 (MRP4)-expressing inside-out vesicles; (**d**–**f**): the inhibition of tracer uptake by unlabeled fluciclovine in (**d**) P-gp-; (**e**) BCRP-; and (**f**) MRP4-expressing inside-out vesicles. Each data point represents the mean ± SD (*n* = 3–6). “ES” and “E2G” indicate “[^3^H]estrone-3-sulfate” and “[^3^H]estradiol-17β-d-glucuronide”, respectively.

[^14^C]Fluciclovine uptake was measured using ABC transporter-expressing vesicles. As shown in Figure 4, no significant difference in [^14^C]fluciclovine uptake was observed between the control, P-gp, and MRP4 vesicles (Figure 4a,c). In contrast, [^14^C]fluciclovine uptake by the BCRP vesicles was reduced in comparison to that of the control vesicles, albeit not significantly (Figure 4b). Furthermore, [^14^C]fluciclovine uptake by the BCRP and MRP4 vesicles showed Michaelis-Menten kinetics (Table 1).

Next, the inhibitory effect of fluciclovine on ABC transporters was also determined using ABC-transporter-expressing vesicles. As shown in Figure 4d–f and Table 1, fluciclovine at millimolar concentrations inhibited P-gp and MRP4, but not BCRP (Figure 4d–f and Table 1). The ABC transporter-mediated transport of specific substrates in the absence of inhibitors was 0.739, 0.523, and 159 pmol/mg protein/min for P-gp, BCRP, and MRP4, respectively.

### 2.5. [^14^C]Fluciclovine Transport via Solute Carrier (SLC) Transporters and the Inhibition of SLC Transporters by Fluciclovine

**Figure 5:**
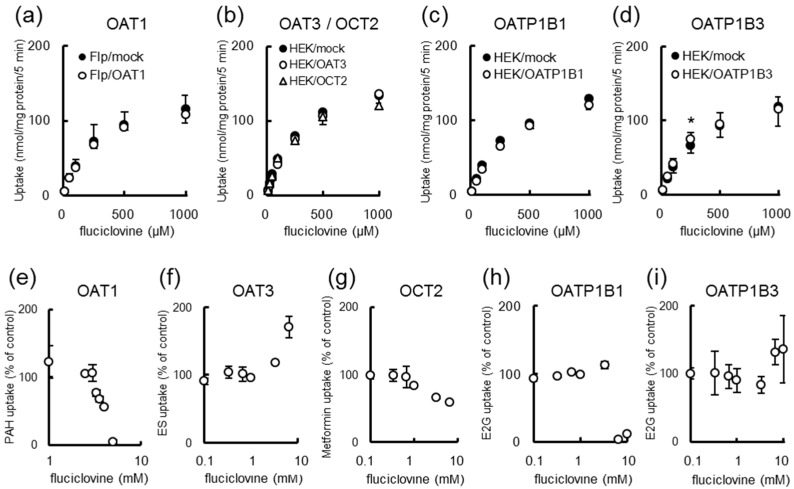
(**a**–**d**): The concentration dependency of the [^14^C]fluciclovine uptake in (**a**) organic anion transporter 1 (OAT1)- (**b**) organic anion transporter 3 (OAT3)-/ organic cation transporter 2 (OCT2)- (**c**) organic anion transporting polypeptide 1B1 (OATP1B1)- and (**d**) organic anion transporting polypeptide 1B3 (OATP1B3)-expressing cells; (**e**–**i**): the inhibition of tracer uptake by unlabeled fluciclovine in (**e**) OAT1-; (**f**) OAT3-; (**g**) OCT2-; (**h**) OATP1B1-; and (**i**) OATP1B3-expressing cells. Each data point represents the mean ± SD (*n* = 3–6); * *p* < 0.05. “PAH”, “ES”, “Metformine” and “E2G” indicate “*p*-[^14^C]aminohippurate”, “[^3^H]estrone-3-sulfate”, “[^14^C]metformin”, and “[^3^H]estradiol-17β-d-glucuronide”, respectively.

Figure 5 shows [^14^C]fluciclovine uptake by SLC drug transporter-expressing cell lines. The intracellular uptake of [^14^C]fluciclovine by mock cells was almost equal to that of OATP1B3-expressing cells, and was higher than that of OAT1-, OAT3-, OCT2-, and OATP1B1-expressing cells (Figure 5a–d). Therefore, the drug transporter-mediated [^14^C]fluciclovine uptake appeared to be negligible. Moreover, [^14^C]fluciclovine uptake in all cells showed Michaelis-Menten kinetics (see Table 1 for the *K_m_* values). Similarly, the inhibition of SLC transporters by fluciclovine was assessed using SLC transporter-expressing cell lines. As shown in Figure 5e–i and Table 1, the inhibition by fluciclovine at millimolar concentrations was observed in OAT1, OCT2, and OATP1B1 cells, but not in OAT3 and OATP1B3 cells (Figure 5e–i). The transporter-mediated uptake using specific substrates in the absence of inhibitors was 38.5, 6.38, 93.5, 3.37, and 0.570 pmol/mg protein/min for OAT1, OAT3, OCT2, OATP1B1, and OATP1B3, respectively.

## 3. Discussion

We conducted a series of studies to assess the involvement of AATs and drug transporters in the distribution of [^14^C]fluciclovine in vitro, thereby specifically focusing on renal reuptake. The apical-to-basal transport of [^14^C]fluciclovine in the LLC-PK1 monolayer model was inhibited by Thr, a substrate representative for system ASC AATs [9,12]. Furthermore, control vesicles/cells and ABC/SLC drug transporter-expressing vesicles/cells showed no significant differences in drug transporter-mediated [^14^C]fluciclovine uptake. Fluciclovine at millimolar concentrations inhibited the uptake of specific substrates by some of the drug transporters tested. Our study suggests that the slow urinary excretion of [^18^F]fluciclovine is partly mediated by system ASC AATs, which serve as high-affinity transporters for [^18^F]fluciclovine uptake/efflux, but that the contribution of drug transporters to [^18^F]fluciclovine distribution is negligible compared to that of AATs.

LLC-PK1 cells acquire cell polarity similar to that of renal proximal tubule cells during monolayer cultivation [21,22]. Regarding the localization of AATs that mediate [^18^F]fluciclovine uptake, ASCT2 (a subtype of system ASC AATs) is localized in the apical membrane of the renal proximal tubule cells [23], and SNAT2 (a subtype of system A AATs) is ubiquitously expressed, including in the kidneys [12,24]. The system L AAT LAT1 is localized both in the apical and basolateral membrane, but its basolateral expression is higher in LLC-PK1 cells [22]. As shown in Figure 3, the contribution of SNAT2 and LAT1 to [^14^C]fluciclovine uptake, which was calculated from the inhibition by MeAIB and BCH, was low at the apical side. These results reveal that system ASC AATs, including ASCT2, at the apical side are the main transporters mediating [^14^C]fluciclovine reuptake by renal proximal tubules. Furthermore, system L in the basolateral membrane may be involved in [^14^C]fluciclovine transport from the renal proximal tubule cells to the blood [22].

System A and ASC AATs recognize l-alanine as a substrate, but its permeation is significantly lower than that of [^14^C]fluciclovine in the LLC-PK1 monolayer model (Figure 2a). If the activity of SNAT2 at the apical side is low as described above, the influx and backflux of fluciclovine may be mediated via system ASC AATs because system ASC AATs function as exchangers [12]. In contrast, l-leucine is one of the substrates of system L AATs, and its permeability profile is similar to that of [^14^C]fluciclovine (Figure 2a). If the involvement of system L is low at the apical side, other AATs present at the apical side (e.g., system B, may be involved in the reabsorption of l-Leu [23]). LLC-PK1 cells are derived from pig kidney; thus, another possibility is that the localization and/or the affinity of amino acids for AATs may be different in other species.

We have previously reported that *p*-aminohippurate (PAH), a substrate for OAT1/3, slightly (approximately 5%) inhibited [^18^F]fluciclovine uptake [8], suggesting that [^18^F]fluciclovine might be anionic at physiological pH and thus might be recognized by anion transporters such as MRP4, OAT1, OAT3, OATP1B1, and OATP1B3 [14]. As shown in Figure 4 and Figure 5 and Table 1, the uptake profiles of [^14^C]fluciclovine fit the Michaelis-Menten kinetics for some drug transporters. Nevertheless, in the present study, there was no significant difference in the [^14^C]fluciclovine uptake between control vesicles/cells and vesicles/cells expressing MRP4, OAT1, OAT3, OATP1B1, or OATP1B3. These observations suggest that AATs, but not drug transporters, are high-affinity transporters involved in [^18^F]fluciclovine uptake, because our previous in vitro study showed that a substrate of system L AATs inhibited [^18^F]fluciclovine uptake to a higher degree than that observed for PAH [8].

As shown in Figure 4b, we observed a difference, although nonsignificant, in [^14^C]fluciclovine uptake by control and BCRP vesicles, suggesting that BCRP might be somewhat involved in [^14^C]fluciclovine transport. BCRP is an efflux transporter that has an important role in drug disposition and distribution, and prevents drugs from penetrating not only the brain and intestines but also tumors, and is also involved in biliary and renal excretion of drugs [14]. Consequently, if the observed difference mentioned above is physiologically relevant, BCRP may play a role in the excretion of [^18^F]fluciclovine from the liver, kidneys, and other organs. In cancer patients, our tracer is not expected to affect the distribution of BCRP-mediated anticancer drugs such as mitoxantrone [25], because here we show that fluciclovine does not inhibit the transport of the BCRP-specific substrate [^3^H]estrone-3-sulfate (ES, Figure 4e and Table 1).

Clinical trials have shown that [^18^F]fluciclovine accumulates in the liver, is excreted slowly in the urine, and provides high tumor-to-normal contrast in cerebral PET imaging because the distribution of [^18^F]fluciclovine to the brain under physiological conditions is restricted [6,26,27]. The major organs targeted by [^18^F]fluciclovine are the liver, kidneys, and brain (the latter via BBB transport) as they express system A (SNAT2), ASC (ASCT2), and L (LAT1) AATs as well as drug transporters [12,14]. However, O’Kane et al. [28] reported that the concentrations of neutral amino acids in the extracellular cerebral fluid are maintained at <10% of those in the plasma by the action of AATs at the BBB. Thus, [^18^F]fluciclovine accumulation in the brain is likely restricted because of neutral amino acid efflux via AATs at the BBB. [^11^C]Methionine, an amino acid PET tracer used in tumor detection, has some properties similar to those of [^18^F]fluciclovine: (1) [^11^C]methionine is transported via AATs [10]; (2) [^11^C]methionine accumulates readily in the liver, moderately in the kidney, but accumulates little in the brain [29]; and (3) [^11^C]methionine accumulation is not affected by P-gp expression [30]. Considering the similarity in the distribution mechanisms underlying [^18^F]fluciclovine and [^11^C]methionine, the present study suggests that, in contrast to that for most therapeutic drugs [31], AATs are the primary contributors to the hepatic, renal, and cerebral biodistribution of [^18^F]fluciclovine.

As shown in Figure 4 and Figure 5 and Table 1, millimolar concentrations of fluciclovine inhibited some of the transporters tested. OAT3-mediated ES uptake increased in the presence of 10 mM fluciclovine, which is in contrast to what was observed for the other transporters tested. A potential explanation for this finding is that the indirect effects, such as a change in the intracellular dicarboxylate concentration, may affect ES uptake because OAT3 functions as an exchanger that exchanges its extracellular substrate and intracellular dicarboxydrate [12]. In contrast, fluciclovine competes directly with the uptake of ES. However, it is unlikely that such inhibition/stimulation will occur clinically because only a small amount of tracer (in the picomolar or nanomolar range) is commonly administered in PET imaging [32]. This, and the fact that [^18^F]fluciclovine is distributed throughout the entire body, prevent the [^18^F]fluciclovine plasma concentration from reaching levels required for inhibition. Therefore, the initial administration of [^18^F]fluciclovine to patients with brain or prostate cancer during PET procedures [2,3] is unlikely to cause drug transporter-mediated drug–drug interactions if generally accepted initial dosages for PET imaging are used.

The glucose analog [^18^F]FDG is a PET tracer that is widely used for cancer diagnosis and, similar to [^18^F]fluciclovine, is not a P-gp substrate [33]. However, treatment guidelines recommend more than four hours of fasting before conducting [^18^F]FDG-PET imaging to avoid drug-food interactions, because high blood glucose levels caused by food ingestion directly affect the tumor-to-normal contrast [4]. In the case of amino acid-derived PET tracers, there is some controversy whether or not short-term fasting is needed to enhance contrast by reducing the plasma amino acid concentration prior to administration of amino acid PET tracers [34,35]. For example, clinical trials of [^18^F]fluciclovine in Japan were conducted with fasted volunteers/patients [2,3,6]. Furthermore, high-protein meals increase plasma levels of free amino acids, including those of branched-chain amino acids [36], which compete for AAT-mediated [^18^F]fluciclovine uptake [9,10]. The present in vitro study indicates that drug transporters are not as crucial for [^18^F]fluciclovine biodistribution as AATs are. Based on treatment guidelines and our present study outcomes, the relationship between food intake and imaging contrast in [^18^F]fluciclovine-PET may warrant evaluation in future studies, because AATs are the predominant transporters involved in the [^18^F]fluciclovine biodistribution.

## 4. Materials and Methods

### 4.1. Reagents and Radioisotope-Labeled Tracers

All reagents were purchased from commercial suppliers (Wako Pure Chemical Industries, Osaka, Japan; Sigma-Aldrich, Japan, Tokyo, Japan; and Nacalai Tesque, Kyoto, Japan) unless indicated otherwise.

In all experiments, ^3^H- or ^14^C-labeled tracers were used because their long half-lives (12.3 and 5700 years, respectively) make them more suitable for in vitro experiments than ^18^F (110 min). *trans*-1-Amino-3-fluoro[1-^14^C]cyclobutanecarboxylic acid ([^14^C]fluciclovine, 2.09 GBq/mmol) was synthesized [9] by Nemoto Science (Tokyo, Japan). l-[^14^C]alanine (5.9 GBq/mmol, for system A and ASC AATs); l-[^14^C]leucine (12.0 GBq/mmol, for system L AATs); d-[1-^3^H(N)]mannitol (455.1 GBq/mmol, for the evaluation of paracellular transport on LLC-PK1 monolayer experiment); [^3^H]digoxin (1104 GBq/mmol, for P-gp); [^3^H]estrone-3-sulfate ([^3^H]ES; 1655 GBq/mmol, for BCRP and OAT3); and *p*-[^14^C]aminohippurate ([^14^C]PAH; 2.04 GBq/mmol, for OAT1) were purchased from PerkinElmer (Waltham, MA, USA). [^3^H]estradiol-17β-d-glucuronide ([^3^H]E2G; 1269 GBq/mmol, for MRP4, OATP1B1, and OATP1B3) and [^14^C]metformin (3.7 GBq/mmol, for OCT2) were purchased from American Radiolabeled Chemicals (St. Louis, MO, USA).

### 4.2. Monolayer Establishment Using LLC-PK1 Cells

The LLC-PK1 cell line was purchased from DS Pharma Biomedical (Osaka, Japan). Cells were maintained in Dulbecco’s modified Eagle’s medium (DMEM; Life Technologies Japan, Tokyo, Japan) supplemented with 10% (*v*/*v*) fetal bovine serum (American Type Culture Collection, Manassas, VA, USA) and antibiotics (100 U/mL penicillin and 100 μg/mL streptomycin (Life Technologies Japan)). Cells were cultured at 37 °C under an atmosphere containing 5% CO_2_ before the start of the experiments.

Cells were seeded at a density of 1.5 × 10^6^ cells/0.25 mL on the membrane of cell culture inserts fitting 24-well plates (upper compartment, apical side; Becton Dickinson, Franklin Lakes, NJ, USA). Flat-bottom 24-well tissue culture plates (Becton Dickinson) containing 0.95 mL of culture medium per well were used as the basolateral side (bottom compartment).

LLC-PK1 monolayer formation was monitored by the measurement of TER using an EVOM-2 and Endohm24 (World Precision Instruments, Inc., Sarasota, FL, USA) according to the manufacturer’s protocol. After cultivation for 4–5 days, the monolayers with a TER value of >250 Ω·cm^2^ were used for the experiment as described in Section 4.3 and Section 4.4. [^3^H]Mannitol transport was measured as described in Section 4.3 for the validation of tight junction formation because mannitol passes through the cell monolayers via the paracellular route.

### 4.3. Apical-to-Basal Transport Using LLC-PK1 Monolayers

Experimental procedures were performed as described previously [37] with minor modifications. After cultivation as described above, the culture media in both compartments were replaced with Na^+^ buffer (137 mM NaCl, 2.7 mM KCl, 8 mM Na_2_HPO_4_, 1.5 mM KH_2_PO_4_, 5.6 mM d-glucose, 0.9 mM CaCl_2_, and 0.5 mM MgCl_2_; pH 7.4 at 37 °C), and the cells were preincubated for 10 min at 37 °C under normal air conditions. After the buffer was removed from each compartment, the permeability experiments were started with the addition of 0.25 mL of Na^+^ buffer containing radiolabeled tracers (final concentration: 81 nM for [^3^H]mannitol and 10 μM for the ^14^C-labeled amino acid tracers) in each upper compartment, and 0.95 mL of Na^+^ buffer in each bottom compartment. The inserts were placed into new 24-well culture plates containing 0.95 mL/well of Na^+^ buffer after 5, 15, 30, 60, and 90 min of incubation. Subsequently, the cells were incubated up to 120 min at 37 °C under normal air conditions.

The apical-to-basal permeability was measured by sampling an aliquot of the buffer from the upper and bottom compartment at the indicated times. To evaluate intracellular accumulation, the cells were lysed in 0.5 mL of 0.1N NaOH at room temperature following rapid washing of the cells with ice-cold Na^+^ buffer. The radioactivity of each aliquot was measured with an LSC6000SC (Beckman Coulter, Inc., Brea, CA, USA) or Tri-Carb 2910TR liquid scintillation counter (PerkinElmer) using Ultima Gold (PerkinElmer).

### 4.4. Inhibition Study Using the LLC-PK1 Monolayer Model

After having developed the LLC-PK1 monolayer model as described in Section 4.2, an apical-to-basal transport study was conducted in Na^+^ buffer (a condition in which all AATs were active) or Na^+^-free buffer (NaCl was replaced with an equivalent concentration of choline chloride, ensuring that only Na^+^-independent AATs were active) containing 10 μM [^14^C]fluciclovine for 120 min at 37 °C under normal air conditions in the presence or absence of 1 mM of the inhibitors. Natural and synthetic amino acids were used as inhibitors [9]: MeAIB (2-(methylamino)-isobutyric acid, for system A in the presence of Na^+^); Thr (l-threonine, for system ASC in the presence of Na^+^); and BCH (2-aminobicyclo[2,2,1]heptane-2-carboxylic acid, for system L in the absence of Na^+^). The apical-to-basal permeability of [^14^C]fluciclovine in Na^+^ buffer without inhibitors was normalized to 100%, and the inhibition of [^14^C]fluciclovine permeation by the natural and synthetic amino acids was calculated as a percentage of the control in Na^+^ buffer.

### 4.5. Uptake and Inhibitory Assay Using ABC Transporter-Expressing Vesicles

For ABC transporters, experimental kits containing Sf9-derived vesicles with high expression levels of human P-gp, MRP4, BCRP, and each ABC transporter (including buffer, ATP/AMP solution, and glutathione) were purchased from GenoMembrane Co., Ltd. (Kanagawa, Japan).

Vesicular transport assays were carried out according to the manufacturer’s protocol. The reaction buffer (50 mM MOPS-Tris, 70 mM KCl, and 7.5 mM MgCl_2_; pH 7.0) contained 4 mM ATP or AMP, unlabeled and labeled fluciclovine, and 2 mM glutathione (only for MRP4). The reaction was started by suspending 50 μg of vesicles (10 μL) in 40 μL of the reaction buffer. After incubation for an appropriate period of time, the reaction was stopped by the addition of ice-cold stop buffer (40 mM MOPS-Tris and 70 mM KCl; pH 7.0), after which the solution was filtered through a 0.45 μm HA filter (Millipore Corp., Billerica, MA, USA) using suction, and was washed twice in 5 mL of ice-cold stop buffer. The radioactivity of the filter was measured with a Tri-Carb 2910TR liquid scintillation counter (PerkinElmer) using Ultima Gold (PerkinElmer). The amount of tracer uptake was normalized to the membrane protein amount described in the product information sheets.

The inhibitory study was performed as described above using appropriate tracers (digoxin: 1 μM, ES: 0.1 μM, or E2G: 10 μM; as described in “4.1 Reagents and radioisotope-labeled tracers”) in the presence of 100 μM–12.5 mM of unlabeled fluciclovine. The tracer uptake amount in the absence of fluciclovine was set as 100%.

### 4.6. Uptake and Inhibitory Assay Using SLC Transporter-Expressing Cells

For the SLC transporter experiments, Mock Flp-in-293 cells (Flp/mock), OAT1-expressing Flp-in-293 cells (Flp/OAT1) [38], mock HEK293 cells (HEK/mock), and OATP1B3-expressing HEK293 cells (HEK/OATP1B3) [39] were obtained from Kanazawa University (Ishikawa, Japan). Briefly, Flp-in-293 and HEK293 cells were transfected with the appropriate plasmid DNA and selected using antibiotics (hygromycin or G-418). All cell lines were cultured in DMEM (Life Technologies Japan) supplemented with 10% (*v*/*v*) fetal bovine serum (American Type Culture Collection) and antibiotics (100 U/mL penicillin and 100 μg/mL streptomycin (Life Technologies Japan) for mock cells, 0.2 mg/mL hygromycin for Flp/OAT1 cells, and 1.2 mg/mL G-418 (Roche Diagnostics K.K., Tokyo, Japan) for HEK/OATP1B3 cells) at 37 °C under an atmosphere containing 5% CO_2_. For the OAT3 and OCT2 experiments, mock HEK293 cells (HEK/mock), OAT3-expressing HEK293 cells (HEK/OAT3), and OCT2-expressing HEK293 cells (HEK/OCT2) were purchased in culture plates from GenoMembrane, Co., Ltd.. For the OATP1B1 experiments, mock HEK293 cells (HEK/mock) and OATP1B1-expressing HEK293 cells (HEK/OATP1B1) were purchased in vials from GenoMembrane, Co., Ltd. All cells were cultured according to the manufacturer’s protocol, and were maintained at 37 °C under an atmosphere containing 5% CO_2_ before the experiments.

The experiments were performed as described previously [39], with minor modifications. Cells provided by Kanazawa University were seeded at a density of 5 × 10^4^ cells per well in poly-d-lysine-coated 24-well flat-bottom tissue culture plates (Becton Dickinson). After 3 days of cultivation, the cells were washed twice with Hank’s balanced salt solution containing Ca^2+^ and Mg^2+^ (HBSS; Life Technologies Japan), after which they were incubated with 0.3 mL of HBSS containing unlabeled and labeled fluciclovine for 5 min at 37 °C under normal air conditions. Tracer uptake was terminated by removing the tracer solution and rapidly washing the cells twice with ice-cold HBSS. The cells were lysed in 0.3 mL of 0.1N NaOH at room temperature, after which the radioactivity of each aliquot was measured as described above. The protein concentration of the cell lysate was determined with a VersaMax microplate reader (Molecular Devices Japan K.K., Osaka, Japan) or SpectraMax i3x (Molecular Devices Japan K.K.; only for OATP1B1) using a BCA Protein Assay Kit (Thermo Fisher Scientific, Kanagawa, Japan). Tracer uptake was expressed as pmol/mg protein. For the cells purchased from GenoMembrane Co., Ltd., the experiments were carried out as described above after cultivation according to the manufacturer’s protocol.

The inhibitory study was performed as described above using appropriate tracers (PAH: 1 μM, ES: 1 μM, metformin: 10 μM, or E2G: 0.1 μM; as described in “4.1 Reagents and radioisotope-labeled tracers”) in the presence of 100 μM–10 mM unlabeled fluciclovine. The tracer uptake amount in the absence of fluciclovine was set as 100%.

### 4.7. Data and Statistical Analysis

All data are presented as the mean ± standard deviation (SD), and are rounded off to three significant figures. *K_m_* and IC_50_ values were calculated using GraphPad Prism 5.0 (GraphPad Software, Inc., La Jolla, CA, USA). Statistical analyses were carried out in the SAS software (version 5.0; SAS Institute Japan, Tokyo, Japan) using two-tailed unpaired *t*-tests or Tukey’s multiple comparison tests. The threshold for significance was *p* < 0.05.

## 5. Conclusions

We show that [^18^F]fluciclovine is recognized as a drug transporter inhibitor with millimolar affinity to the tested drug transporters in vitro, suggesting that slow urinary excretion of [^18^F]fluciclovine is mediated in part by system ASC AATs, but not by drug transporters.

## Figures and Tables

**Table 1:** *K_m_* and IC_50_ values of fluciclovine for specific drug transporters.

Transporters	*K_m_* (mM)	IC_50_ (mM)
Efflux Transporters		
P-gp	N.D.	2.95 ± 2.56
BCRP ^1^	1.34 ± 1.45	N.D.
MRP4 ^1^	15.3 ± 44.1	9.28 ± 1.90
Uptake transporters		
OAT1 ^2^	258 ± 48.2	3.98 ± 1.77
OAT3 ^3^	N.D.	
OCT2 ^3^	275 ± 36.6	8.95 ± 1.96
OATP1B1 ^4^	401 ± 41.0	5.41 ± 2.49
OATP1B3 ^5^	238 ± 82.5	N.D.

All values represent the mean ± SD (*n* = 3–6). N.D.: Not determined within the concentration range used in this study. ^1^ Values at all points were not significant in comparison with those of control vesicles, but *K_m_* is shown because these data fit Michaelis–Menten kinetics; ^2–5^ The calculated *K_m_* values (μM) of the mock cells were 258 ± 127 (*n* = 6), 340 ± 43.1 (*n* = 3), 349 ± 40.0 (*n* = 3), and 340 ± 161 (*n* = 6). P-gp: P-glycoprotein; BCRP: breast cancer resistance protein; MRP4: multidrug resistance-associated protein 4; OAT1: organic anion transporter 1; OAT3: organic anion transporter 3; OCT2: organic cation transporter 2; OATP1B1: organic anion transporting polypeptide 1B1; OATP1B3: organic anion transporting polypeptide 1B3.
